# A randomized EPIREMED protocol study on the long-term visuo spatial effects of very preterm children with a working memory deficit

**DOI:** 10.1186/s12887-021-02867-x

**Published:** 2021-09-13

**Authors:** Catherine Gire, Any Beltran Anzola, Monique Kaminski, Karine Baumstarck, Pierre-Yves Ancel, Julie Berbis, for EPIREMED-Study Group, Meriem Zahed, Patricia Garcia, Tristan Desiles, Ludovic Zahed, Mélodie Pache, Gwenaëlle Menard, Nathalie Bednarek Weirauch, Karine Voirin, Virginie Verriere, Gilles Cambonie, Claire Lerat, Maythé Poujol, Olivier Claris, Sophie Rubio Gurung, Eliane Basson, Melanie Rodriguez, Anne Rannaud, Johanna Boulant, Thierry Debillon, Isabelle Pin, Karine Guichardet, Caroline Tournegros, Laurence Foix L’Helias, Delphine Mitanchez, Jennifer Sommer, Hélène Ruys Masson, Michele Granier, Marylène Riou, Dalia Mignot, Bernard Guillois, Valérie Dorriere Datin, Mireille Denaveaut Boulay, Delphine Rots, Jean-Michel Hascot, Hélène Deforge, Sabine Guignon, Pierre Kuhn, Anne de Saint Martin, Claire Zores Koenig, Hélène Musmeaux, Lucille Schneider, Carole Ramousset, Coralie Mangin, Bénédicte Lecomte, Angélique Pannetier, Emmanuelle Rochette, Nelly Goudon-Dubois, Julie Oertel, Sandrine La Planeta, Stéphane Marret, Marie Lemarchand, Nathalie Mestre, Hugues Patural, Sophie Flori, Jean-Christophe Roze, Charlotte Coudronniere, Hamida Martin, Alix Laurent, Elie Saliba, Patrick Zander, Eva Aoustin, Catherine Arnaud, Emeline Dubois, Stephanie Iannuzzi, Carine Duffaut, Isabelle Souksi Medioni, Magali Rebattel, Elodie Falque, Nathalie Rumeau, Valérie Benhammou, Laetitia Marchand-Martin, Samira Medjahed

**Affiliations:** 1 Department of Neonatology, North Hospital, APHM University Hospital, Marseille, France; 2 CEReSS - Health Service Research and Quality of Life Center, Faculty of Medicine, Aix-Marseille University, 27 Bd Jean Moulin, 13385 Marseille, cedex 05 France; 3 University of Paris, CRESS, Obstetrical Perinatal and Pediatric Epidemiology Research Team, EPOPé, INSERM, INRAE, F-75004 Paris, France; 4 Obstetrical, Perinatal, and Pediatric Epidemiology Team, Center of Research in Epidemiology and Statistics (U1153), Paris University, INSERM, Paris, France; 5 Clinical Research Unit, Center for Clinical Investigation P1419, CHU Cochin Broca Hôtel-Dieu, Paris, France

**Keywords:** Very preterm children, Working memory, Visuospatial index, Cognitive training, Executive functions

## Abstract

**Background:**

Very preterm children generally perform poorly in executive functions and particularly in working memory. Adaptive training tasks encouraging these children to work continuously on their personal working memory capacity can be very useful. Above all in preschool-age children, several cognitive training programs focused on improving working memory capacity. Cogmed is a computerized visuospatial cognitive training program that improves working memory in children and adolescents with attention-deficit/hyperactivity disorder. The main objective is to assess the long-term effects (18 months) of cognitive training (Cogmed) on visuospatial processing in preschool-age very preterm children with working memory impairment.

**Methods:**

The EPIREMED study is a prospective, randomized, controlled, multicentric trial nested in a population based epidemiological survey. An intervention group (Cogmed cognitive training) and a control group (standard care management) will compare children aged 5½ to 6 years, born between 24- and 34-weeks’ gestational age, with a global intelligence quotient > 70 and a working memory index < 85. The study will include 166 children from national study EPIPAGE-2 (Epidemiological Study on Small Gestational Ages). The intervention consists of 25 sessions administered over a 5- to 8-week period. The primary endpoint will be the visuospatial processing, assessed by the score of the visuospatial index: score of the WPPSI-IV (Wechsler Preschool and Primary Scale of Intelligence). The secondary endpoints will allow to assess the executive functions, language and abilities, infant behavior, quality of life assessment, school performance and parental anxiety.

**Discussion:**

This project’s primary goal is to demonstrate the necessity of early visuospatial memory assessment within the vulnerable population of very preterm children, and to prove the feasibility and efficacy of computerized cognitive training using online software programs. A better global neuropsychological development improvement (visuospatial processing and other far transfer) can be expected with an improvement in learning and decreased behavioral problems. In the long term, these improvements might also reduce those global costs linked to the consequences of extreme prematurity.

**Trial registration:**

NCT02757794 (registered on 2nd May 2016 at ClinicalTrial.gov).

## Background

Very Preterm (VP) birth rates and survival rates have risen in France [1, 2]. However, the proportion of VP survivors with severe deficits has remained stable. Neuropsychological disorders and/or behavioral deficits are the most frequently encountered deficiencies [2–8] and have serious consequences on learning [9], familial and social adaptations as well as an impact on the child’s future adult years [10].

### Executive function

Executive Functions define the cognitive operations that allow an individual to adjust behavior and activity in response to environmental requirements and fluctuations.

Compared to peers born at term, VP children generally perform poorly in Executive Functions (EF) and, in particular, in Working Memory (WM).

The EF comes into play when an individual is confronted with a “non-routine” situation which requires problem solving. The principal mental processes characterizing EF are:

- organizing and planning data based on what needs to be achieved, choosing relevant information,
- implementing processing operations, inventing new situations and modifying them if they deviate from the (original) purpose [11, 12],
- suppressing extraneous information, resisting distractions,
- organizing task-relevant information in their memory for later use (WM).

While each of these mental processes or “executive mental functions” can be evaluated by “specific” tests, they are often interlinked and dependent on the mental attention processes (auditory and/or visual). For this reason, definitions vary significantly from publication to publication [11, 13].

Schematically we can isolate four main executive mental processes [14]:

1. Planning: mental diagram of a single action, anticipating the goal to be reached - “how to achieve that goal”.
2. Flexibility: adaptation of an action plan for environmental contingencies, ability to modify strategies in case of error, maintain attention - “the art of adapting to change”.
3. Inhibition: capacity to ignore distractions and to resist giving one reply rather than another.
4. Working Memory: ability to store verbal or visuospatial information in one’s mental space and to manipulate it, implementing strategies, processing action sequences, reasoning - “*art de faire*”. (Having the knack)

### Executive function and very preterm

There is a considerable interest in EF in very preterm children [15, 16]. In studies published between 1990 and 2008 a systematic review performed by Mulder et al., analyzed the executive process in 830 very prematurely born school children vs 740 children born full-term [15]. The authors showed a difference of 0.3 Standard Deviation (SD) for inhibition, 0.5 SD for verbal Working Memory (WM) and 0.4 SD for planning skills as compared to the full-term control group. These differences were even greater for those with lesser gestational ages: 0.5 SD for the inhibition and 0.7 SD for verbal WM within 26 weeks GA (weeks’ amenorrhea).

This gap is interesting, especially for verbal WM, and it increases with the age of children suggesting a worsening over time. Mental flexibility remains unchanged as compared to the control group. The Aarnoudse-Moens’s meta-analysis involved children born between 1998 and 2008 comparing very extremely premature births to full-term births [16]. This meta-analysis showed a decrease of 0.6 SD for verbal WM and 0.4 SD for the visuospatial WM. Unlike Mulder et al., this difference is shown to stabilize with age, and their mental flexibility is impacted 0.5 SD as compared with the control group.

In 2018–2019, Brydges’ meta-analysis of 60 studies included a total of 6163 preterm children born before 32 weeks’ amenorrhea and a control group of 5471 full-term children. The children in both groups were between 4 and 17 years of age [17]. Altogether, VP children obtained a result of − 0.51 SD (95% CI 0.44–0.58; *p* < 0.001) for executive functions. Van Houdt, in 2019, compiled 35 studies (3360 preterm and 2812 full-term infants) and reported an overall deficit in EFs in VP children of approximately − 0.5 SD [18]. The study observed a deficit of − 0.52 SD in working memory, − 0.39 SD in inhibitory control, and − 0.51 SD in flexibility. However, the deficit difference in each domain was not significant. All three EFs are affected to the same extent in VP children.

These data are corroborated in the meta-analyses of Twilhaar in 2018 [19] regarding academic performance (17 studies, 2390 children, − 0.71 SD in mathematics, − 0.44 SD in reading and − 0.52 SD in spelling). Allotey in 2018 reported − 0.67 SD in reading, − 0.56 SD in spelling and − 0.78 SD in mathematics amongst VP children aged 5-to-8- years [6]. Additionally, Allotey pointed out that this difference in academic results continued to persist in secondary education. The latest meta-analysis of 33 studies, which covered 4000 premature infants, confirms this academic gap in math and reading performances between VP children and their peers [20]. Two meta-analyses looked at the evolving profile of executive deficit in extremely premature infants [18, 21]. The last, by van Houdt, suggested a stability of this deficit, at least over studied ages of 4½ and 14 years of age. The authors do not exclude possible modifications afterwards, at the end of adolescence or in adulthood, in view of the slow maturation of EFs.

### Working memory

WM is defined as a “brain system that provides temporary storage and manipulation of information necessary for complex cognitive tasks” [22]. WM is regulated by a central executive control system and two subordinate subsystems: the visuospatial sketchpad and the phonological loop. This process is considered as a prerequisite for other EFs such as reasoning and planning and for predicting intelligence and academic success. In VP children, WM impairment is linked to learning disabilities and is reported to have a strong influence on language and visuospatial processing [23]. Very premature-birth children present with frequent WM deficits [16, 24, 25]. Indeed, the literature shows that visuospatial WM is not as good in VP preschool children as term birth children [26–28]. In 2014, Omizollo et al., studied the correlation between WM (verbal and visuospatial) and the learning in a cohort group of seven-year-olds very prematurely born. The VP group had 2.1 to 3.5 times more deficit in WM than the control group [29]. In these school age children, WM might be correlated with subsequent learning disorders and the origin of complex deficits such as language delays or visuospatial performance disorders [15]. Overall academic achievements are thus impaired and an intervention strategy to minimize prematurity’s long-term WM impact needs to be developed.

### Cognitive training

In recent years, several Cognitive Training (CT) programs have focused on improving WM capacity by adaptive training tasks that encourage individuals to work continuously on their personal working memory capacity. Many re-education techniques (books, games, software, etc.) have emerged in recent years without any “gold standard”. Cogmed JM [30–32], for pre-schoolers ages four to six, is a technique for re-educating the visual spatial WM and is used in many randomized studies of CTs, WMs and EFs [33]. This computer software has a specific set of visuospatial memory tasks and adjustable levels of difficulty by using a precise algorithm. These programs have succeeded in improving individual performance in some specific WM capacities, but not in other everyday EF functions such as, language and visuospatial processing [11, 34, 35]. Therefore, the functional benefits of CT have become controversial. Recently one metanalysis showed that cognitive training programs for preschoolers are significantly more effective for developmentally at-risk children (ADHD or low socio-economic status) than for children with typical development and without risks. This metanalysis assessed also other factors as the individual vs collective session and the training duration; these two factors were considered significant moderators contrary to the number of sessions and the computerized against non-computerized training [36]. The efficacy of Cogmed was particularly studied by two scientific publications, Shinaver et al. and Spencer-Smith et al. [33, 37]. The impact on the trained WM visual spatial is significant and seems to be sustained over time. Improving non-trained functions such as verbal WM, attention, and secondary learning disorders, are possible, but have yet to be proven on larger numbers. Above all the WM cognitive training studies of preschool age children were realized with Cogmed JM [30–32].

### Cognitive training and prematurity

Three studies on premature infants have examined the effects of CT using Cogmed on visuospatial WM [38–40]. The first was a sample of 16 preterm vs. 19 term-birth teenagers. The second was performed on 20 VP preschool children ages 5–6 years and the third study included 20 VP preschool children vs 17 term births.

There appears to be a beneficial effect on the trained visuospatial WM and a possible transfer to other processes (verbal working memory, attention, etc.). However, these three studies contained very small samples from very different age groups. In two studies the WM was in the process of maturation, and in the third it was practically consolidated. Although, monitoring was limited to 7 months and learning disorders were not assessed, they nonetheless represented preliminary studies with encouraging results.

The IMPRINT study [41, 42] is the only large, double-blind, randomized study using a Cogmed intervention group and a control group (Cogmed placebo without TM training). The study includes 126 seven-year-old, VP children (< 28 SA, < 1000 g, with no cognitive deficits, and having a median IQ 95). The IMPRINT study shows that only the working memory at 2 weeks post intervention was improved in the intervention arm. This did not persist at the first and second year after training. There was no benefit from the Cogmed training on their executive behaviour, their attention or their school results, either at 2 weeks after the intervention or after 1 to 2 years after the intervention.

The authors concluded that Cogmed was not recommended for improving academic performances for VPs at this age (7 years) [42]. Thus, it seems that for VP children, the Cogmed computerized WM training program can provide an immediate and direct WM benefit but no immediate transfer and no long-term benefits.

As executive disorders are the core of VP neurodevelopmental problems (learning difficulties, attention disorders, behavioral disorders) and are identified in VP children, it raises the question of possible cognitive training of executive functions to reduce problems and improve the daily quality of life for the children and their parents. Once the concept of executive training is put forward, the questions are: the temporality of the training and at what age will this executive training be most relevant? What are the precise objectives of this training and how will it be evaluated? Finally, the precise modalities (duration, type of exercise, chosen medium, etc.) of the training program need to be defined to maximize its effectiveness [43].

### Hypothesis

Even if some literature data exist on Cogmed’s CT visuospatial WM effects in premature infants there are some limits that need to be discussed. The sample size and compliance of training from participants might influence the robustness of the results. In fact, those elements of these studies are generally modest (below or about 50 patients randomized in interventional group and around 20 patients with good training compliance) [38–42]. Furthermore, none of these studies have been carried out in France where the aspects of the French healthcare system are very specific. Moreover, the efficacy of visuospatial CT might be modified according to the age at which children perform the CT. In fact, visuospatial CT is a well-adapted method to reach WM in children 5-to-6-years old whose WM is only a visuospatial (subcomponent visuospatial sketchpad), taking advantage of the neuroplasticity period. The central executive control system and the phonological loop will develop in children later on. Furthermore about 30% of VP children have low performances on their visuospatial processing. Thus, a visuospatial CT for 5-to-6-year olds will enable a measurement of the consequences of visual spatial WM restoration on the overall brain function and learning in VP children [38, 39].

We hypothesize that CT with preschool VP children (just as their WM is emerging and uniquely non-verbal: visuospatial sketchpad) may decrease the subsequent dysexecutive disorders, improve intellectual performance (both visuospatial and language processing) as well as school integration. This study will therefore assess CT ability to improve visuospatial processing and moreover to assess its impact on the global function and learning abilities of the brain.

### Objectives

Our primary objective is to assess the long-term effects (18-months post inclusion [+/− 2 months]) of Cognitive Training (Cogmed JM) on the visual-spatial processing in VP preschool-aged children with WM impairment. Visuospatial processing is a broad cognitive process encompassing many subcomponents such as attention, sensory-motor skills, EF and visuospatial WM.

The secondary objectives are to assess the effects of the cognitive training on the following parameters at the six-month post-intervention (+/− 2 months):

Children’ parameters:

- Global intellectual functioning,
- different cognitive processes: working memory,
- language, visual-spatial processing, speed processing,, and fluidity of intelligence,
- other composites of executive functions: auditory attention, flexibility, and inhibition,
- language processing abilities: verbal learning abilities (cultural and cognitive), phonological judgment and semantics, verbal processing speed, verbal WM, motor programming, visual attention, and ability to analogize,
- behavior and quality of life (QoL),
- school performance.

Parents’ parameters:

- Anxiety level.

## Methods/design

### Study design

This study is designed as a multicenter (18 units of French university hospitals), randomized, controlled, open-label, two-parallel groups study. The recruitment will be prospective. The two groups are:

- A control group: standard care management,
- an experimental group: standard care management in association with a 2-month Cognitive Training program called Cogmed JM.

The list of the recruiting centers is availed in website *clinicaltrial.gov*

### Participants

Participants must meet all of the following criteria:

#### Inclusion criteria

- children already included in the EPIPAGE 2, born between 24-and 34-weeks’ gestational amenorrhea,
- children aged 5½ to 6 years old,
- children exhibiting a total intellectual quotient > 70 from the Wechsler Pre-Primary Scale of Intelligence – Fourth Edition (WPPSI-IV) (during the 5-year assessment in EPIPAGE 2),
- children having a visuospatial WM impairment defined by a working memory index <= 85 from the WPPSI-IV,
- children with parents (or legal guardians) authorizing participation in the study and a signed informed consent form,
- children with medical insurance.

#### Non-inclusion criteria

- Children with severe cerebral palsy, based on the Gross Motor Function (score > 2) and Bimanual Fine Motor Function (score >) 2 classification system [44, 45],
- children with blindness or amblyopia, defined by a visual acuity < 3 (during the 5-year assessment in EPIPAGE 2),
- children with deafness, as defined by a prescribed hearing aid,
- children with chromosomal disorder or autistic syndrome,
- children included in the EPILANG study protocol (VP children with language delay and parent intervention [Effectiveness of speech-language pathology parental guidance for very preterm infants with language delay] (an ancillary project to EPIPAGE 2),
- children who do not speak French,
- children with parents having no internet connection,
- triplets.

#### Exclusion criteria

- Children and / or parents wishing to interrupt his / her participation during the study.

### Groups

#### Experimental group: the cognitive training (CT)

A neuropsychologist or speech therapist will oversee the experimental group’s Cogmed training, and build a support structure for both the patient and their parents during the initial interview. Children and their parents will become acquainted with the program which will include a software presentation, the setting of expectations, the CT objectives and establishing a reward system along with a document to explain WM and the software. The program includes a total of three 15-min sessions per week for 8 weeks and involves.

Cogmed JM (4–7-year-olds) is a computerized, online WM rehabilitation program. This will be executed at home, at the hospital or in a rehabilitation center with a “tutor” according to the parents’ abilities and their access to an internet connected computer. The child, who will be accompanied by either one or both parents or by a “guardian”, will be given a series of interactive, automatically and individually adapted exercises. In Cogmed JM, sessions last 15–20 min, with three exercises out of seven for each session, with a graphic interface.

The Cogmed JM practitioner will consult the on-line compliance and exercise results after each session. The program calculates the performance index: the difference between the maximum level and the starting level which is used to assess progress against a standard norm.

The parents will receive a weekly, 30-min interview in order to support and strengthen their child’s motivation. The interview will focus on the child’s evolving performance and reward progress.

#### Control group: current standard care management

The control group will not be offered a rehabilitation program but rather be followed-up along with their routine care management. Speech therapy and/or academic support may be recommended for those experiencing scholastic difficulties. The control group’s visits and questionnaires will be the same as for the parents and the teacher in the experimental group.

### Endpoints

#### Primary endpoint

The visual-spatial processing will be assessed by using the visuospatial index (VSI) of the WPPSI-IV at the inclusion visit, the six-month post-inclusion (+/− 2 months) and at 18-months post inclusion (+/− 2 months). This index consists of two subtests: block design and object assembly. The average score is 100 with a standard deviation of 15.

##### Rationale for the primary endpoint

The visual perceptual integration testing performance for VPs is poorer than those born full term, and these VPs present with a visuo-constructive dyspraxia. This deficit is connected to a poor integration of visual function, perceptual and/or fine motor skills (e.g., reproduction of a complex geometric figure). This disorder is three times more frequent in VP adolescents than in those born at term. Among those born extremely prematurely, 30% have results below the 15th percentile in visuospatial performance [46]. The impact of WM rehabilitation on visuospatial skills is an interesting line of research, most particularly in premature infants.

#### Secondary endpoints

The secondary endpoints are related to the children and to their parents will be assessed at the inclusion visit and at the six-month post-inclusion (+/− 2 months).

##### Children’s endpoints

- The intellectual functioning and other cognitive processes will be obtained by global intellectual quotient (IQ) and IQ indexes using the WPPSI-IV (Wechsler) [47].

The WPPSI-IV, designed for children ages 4-to-7-years-old, assesses the overall intellectual functioning (total comprehensive intellectual quotient) and specific cognitive processes. This is done through the main indices:

- verbal comprehension index,
- fluid reasoning index,
- visual spatial index,
- processing speed index, and
- the working memory index.

The evaluation of the Working Memory Index by the WMI in WPPSI-IV is uniquely visuospatial at this age. The average is 100 with a standard deviation of 15, as with all Wechsler Scales. The global IQ, as well as the main indices, will be assessed when monitoring the EPIPAGE cohort. It is lower for VP infants as compared to full-term infants [5].

- Executive and attention processes: The NEPSY-2 (NEuroPSYchology assessment - Second Edition) assesses the neuropsychological development of preschool and school age children (3–12 years old) and is used to obtain emerging executive functions in 5–6-year-olds [48]. Auditory attention, statue, and design fluency are the only three subtests in the Executive and Attention Function domains which will be administered in this study. These three tests measure selective and divided attention in auditory modality, inhibition and mental flexibility. A review of Van de Weijer-Bergsma, which is confirmed by Mulder in a meta-analysis, shows that the selective, divided and supported attentional domains are likely to be affected in VP pre-schoolers [15, 49].
- Evaluation of language and its skills: Language is a complex mental process requiring an assessment of all its components. This assessment will be made from the CLéA battery calibrated for those children between 2 and 15-years-old [50].
  The battery consists of seven tests:
  - Known Digital Channels: a reflection of verbal learning ability, both cultural and cognitive.
  - Oral Word Identification: capacity to give a word phonological and semantic judgment.
  - Rapid Denomination: timed tested; gives an indication treatment speed.
  - Word memory: explores the short-term mnemonic span.
  - Facial and oral praxis photographs: relevant in motor programming.
  - Visual attention: inspired from NEPSY, it questions the visual spatial component.
  - Resolution of logical problems: ability to reason analogically (progressive matrices).

Studies suggest that specific language disorders can be associated with specific WM impairments, particularly with the phonological loop [51, 52]. Two metanalyses-studies demonstrate global language gaps/impairment in the very premature vs. the full-term child with deficits in learning ability, phonology, semantics, grammar, speech coherence and verbal reasoning [53, 54].

- Behavioral Evaluation: the child’s behavior will be assessed with the Goodman Strengths and Difficulties Questionnaire, which includes 25 self-administered questions answered by the parents [55], and will assess any impact this intervention has on the child’s behavior.
- Evaluation of the child’s quality of life (QoL): The quality of life of the children will be assessed using the Perceived Quality of Life and Health of Adolescents and Children Questionnaire (VSP-A [*Vie et Santé Perçue de l’Adolescent et de l’enfant*]) [56] as reported by the parents. The 49-item version portrays nine dimensions and index:
  - relationships with parents/family,
  - body image,
  - vitality,
  - relationships with friends,
  - general well-being,
  - leisure,
  - school performance,
  - relationships with teacher,
  - relationships with medical staff.
  A higher score, which ranges between 0 and 100, indicating a better QoL. The French norms are available through Ravens-Sieberer U 2007 [57].
- Schooling: This will be evaluated by the GSA questionnaire (Global School Adaptation score), a French tool completed and validated by the teacher, [58] and re-evaluated in a preschool population in 2013 [59]. The questionnaire covers five verbal skills (verbal communication, verbal participation, vocabulary, syntax, pronunciation), five non-verbal abilities (memory, arithmetic, logical reasoning skills, manual dexterity and fine motor skills) and eight questions evaluating class behavior (compliance with rules, attention, autonomy, speed of accomplishing the task, self-esteem, ability to keep the pace and fatigability). The final question asks the teacher about possible future special educational needs of the child.

##### Parents’ endpoints

- Anxiety: The Spielberger state-trait anxiety inventory (STAI) will be used to assess anxiety. The STAI is a self-reporting questionnaire consisting of 40 items that measure both the state and trait scores. These scores range from 20 (absence of anxiety) to 80 (high anxiety) [60]. This questionnaire will assess if anxiety is impacted as a result of parental intervention (mother).

##### Rationale for the secondary endpoints

It is of value to measure the impact of intervention on the WM and on other non-trained brain processes as well as on parental anxiety, child behavior and parental perceptions of the child’s quality of life.

### Participant timeline

**Fig. 1:**
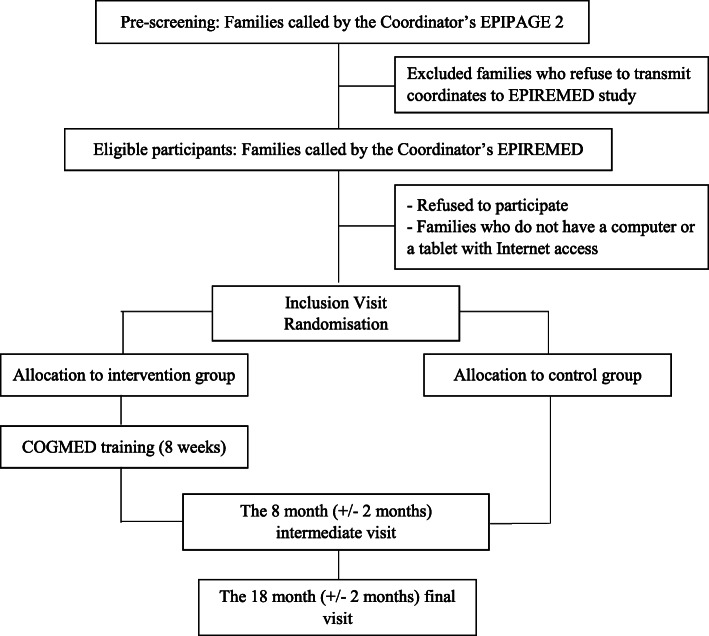
Flowchart EPIREMED Study

The total participation time is 20 months (Fig. 1).

### Sample size

The sample size was calculated to obtain an 80% power to detect a difference of 7.5 points on the VSI index (estimated standard deviation: 15) at 18 months (+/− 2 months) between the two groups. This has been considered to be clinically significant considering other similar studies [5]. With the threshold for statistical significance set at a *p*-value of 0.05, assuming that a potential 15% of patients will be lost to follow-up between baseline and last assessments, these calculations showed that 166 patients are needed (83 per group [Power Analysis and Sample Size Software Version 2008, Utah, USA]). Assuming that 30–40% of the EPIPAGE 2 children will present with a WM abnormality, 1600 children will be required for screening.

### Recruitment

The study will include children in EPIPAGE 2 from the regions participating in the present study (EPIREMED). Recruitment will be at the end of the EPIPAGE 2 assessment (5 ½ years). The EPIPAGE 2 protocol and the main perinatal results are described in two publications of Ancel P-Y et al. [2, 61].

The EPIPAGE 2 clinical research coordinator will establish a monthly, per center list of potentially eligible patients to EPIREMED. Using this list, each center’s referring staff person will organize the first telephone interface with those qualified families having a WM index lower than 85, who have no exclusion criteria and who have the ability to travel to the testing centers.

Thereafter, the coordinator will contact those families to further coordinate their EPIREMED participation. The coordinator will explain the study’s objectives, its development, and its advantages and disadvantages.

Those families accepting to participate will receive a parent information letter describing all topics discussed by telephone, an information text specifically adapted for a child, an informed consent and a verification of their appointment. Travel expenses will be reimbursed.

### Randomization

A randomization list will be established before the implementation of the study with a 1:1 allocation ratio, and will be elaborated by a secure Clinical Research Platform. A computer-generated, randomized list using a permuted block design will be done (stratified on center and gemellarity: singleton/twin). Multiple births represent 30% of the preterm population within the EPIPAGE 2 group. If one twin has a WM anomaly, this child will be selected for randomization.

### Data collection methods

#### The inclusion visit

After consents have been obtained from the parent or legal guardian, the following assessment will be obtained:

- The children
  Assessment of the working memory index (WMI)
  Auditory attention, statue, and design fluency of the NEPSY-2
  Language and verbal skills (CLéA)

This assessment will be made by a neuropsychologist and will last about 90 minutes.

b) The parents:
  A self-administered questionnaire on anxiety (STAI)
  A questionnaire assessing the behavior of the child (Goodman)
  A questionnaire assessing the child’s QoL (VSP-A)
c) The teachers:
  A questionnaire assessing the School Adaptation (GSA questionnaire)

The Cogmed JM group will begin no later than 2 months after the participants’ inclusion and the standard group will receive routine management. A similar follow up will be planned for the two groups, based on their intermediate and final visits.

#### The 8 month (+/− 2 months) intermediate visit

An assessment will be conducted 8 months (+/− 2 months) after the participants are included in the study and will consist of:

- The children
  Assessment of the working memory index (WMI)
  IQ and its main indices (WPSSI IV)
  Auditory attention, statue, and design fluency of the NEPSY-2
  Language and verbal skills (CLéA)

This assessment will be made by a neuropsychologist and will last about 180 minutes.

b) The parents:
  A self-administered questionnaire on anxiety (STAI)
  A questionnaire assessing the behavior of the child (Goodman)
  A questionnaire assessing the child’s QoL (VSP-A)
c) The teachers:
  A questionnaire assessing the School Adaptation (GSA questionnaire)

#### The 18 month (+/− 2 months) final visit

Children in both the intervention and control groups will receive a final 18-month evaluation (+/− 2 months) after inclusion; they will then be between 7 and 7½ years old.

The primary evaluation end point: The visuospatial index and the working memory index of the WPPSI-IV will be used for this visit. The WMI will be used to determine the long-term maintenance of the working memory and its impact on intelligence. Auditory attention of NEPSY-2 will be used in this final evaluation. A neuropsychologist will make the assessment that will last about 30 min.

### Data management

Using EpiData software, a specific database will be created. Each participant will be assigned a unique, anonymous number and a data quality control will be performed by a physician to minimize any data inconsistencies.

### Statistical methods

Data will be analyzed using the Statistical Package for the Social Sciences (SPSS) version 20.0 software. Statistical significance is defined as *p* < 0.05. The methodology will be based on the Consolidated Standards of Reporting Trials Statement (CONSORT, http:// www.consort-statement.org/consort-statement/) [62].

The full analysis set will be used for the primary analysis. There is no interim analysis planned.

Demographic and baseline characteristics will be summarized. Quantitative data will be shown as mean ± standard deviation or median with its interquartile range. The qualitative data will be described as percentages with a 95% confidence interval.

#### Analysis of primary endpoint

The mean scores of the VSI will be compared between groups (Student t test or Mann Whitney test). Linear regression will be performed to adjust for potential confounding factors; variables relevant to the models will be selected on their clinical interest and/or a threshold *p*-value <= 0.1 during bivariate analysis. The final models will express the beta standardized. The unadjusted analysis will be the primary analysis, and the adjusted analysis will be secondary analysis.

##### Methodology to account for missing data

Children not meeting the primary endpoint measurements will be considered as having failed in the study regardless of their randomization group. Additional analyses will be conducted based on:

- Available data and,
- After multiple imputation of missing data.

#### Analysis of secondary endpoints

The secondary endpoints will be compared between groups according to the nature of the variable.

The analysis for repeated measurements will be performed to compare changes over time (baseline, eight (+/− 2 months) and 18 months (+/− two months) between groups.

##### Patient selection to include in the analysis

All the children registered and randomized in the study will be included in the analysis, respecting the intention to treat. This analysis will be conducted, if necessary, under the maximum bias hypothesis. In a second step, those wrongly included, and any major protocol deviations, will be excluded from the analysis.

## Discussion

This project’s primary goal is to demonstrate the necessity of early visuospatial memory assessment within the vulnerable population of VP children, as well as to prove the feasibility and efficacy of computerized CT using online software programs.

Neurodevelopmental problems are common in VP children. Recent publications have reported many disorders in their specific cognitive functions, one of which is WM. Although neuro-development of VP children remains a public health priority because of their increased birth and survival rates, the interventions to improve this are few. There are currently no truly effective interventions to deal with academic achievement in preschool-age VP infants and their neuropsychological problems; thus, currently no specific management care is recommended for these children.

Cogmed is based on interactive software and parental support. The program takes into account the child’s environment, a factor of great relevance since it is closely linked to the child’s future development. The expected benefits of CT (Cogmed) for those children in this study are enhanced WM, possibly leading to better EF by taking advantage of cerebral plasticity.

A better global neuropsychological development improvement (language and visuospatial processing) can be expected with an improvement in learning and decreased behavioral problems. For parents their guidance in Cogmed helps reduce their anxiety by fully embracing their role as primary agents in their child’s development. This is consistent with recommendations for family-centered healthcare and can significantly improve the quality of life for both the children and their parents. In the long term, these improvements might also reduce those global costs linked to the consequences of extreme prematurity.

Finally, if proved effective for this vulnerable population, this treatment can be a possible option or an alternative for other preschool populations complaining of early academic difficulties related to WM deficits. Furthermore, the study design based in a randomized, placebo-controlled, is considered as an appropriate design to demonstrate the efficacy of a new experimental intervention in accordance with the Levels of Evidence classification of the Evidence-Based Medicine Working Group [63]. Our findings could be used in the future to update the national and international recommendations concerning very preterm children and training of executive functions.

## Data Availability

The datasets that will be generated and/or analyzed during the current study are not publicly available due to the data belongs to the Assistance Publique Hopitaux de Marseille. However, datasets are available from the sponsor (promotion.interne@ap-hm.fr) on reasonable request and after sign a contract pertaining to the provision of data and /or results.

## Abbreviations

CT: Cognitive Training
EF: Executive Functions
EPIPAGE: Epidemiological study on small gestational ages
GSA: Global School Adaptation score
IQ: Intellectual Quotient
NEPSY-2: NEeuroPSYchology assessment – Second Edition
QoL: Quality of Life
SD: Standard Deviation
STAI: Spielberger State-Trait Anxiety Inventory
VP: Very Preterm
VSI: VisuoSpatial Index
VSP-A: Perceived quality of life and health of adolescents and children questionnaire
WM: Working Memory
WMI: Working Memory Index
WPPSI-IV: Wechsler Pre-Primary Scale of Intelligence – Fourth Edition
